# Using a gamified monitoring app to change adolescents’ snack intake: the development of the REWARD app and evaluation design

**DOI:** 10.1186/s12889-016-3286-4

**Published:** 2016-08-05

**Authors:** W. Van Lippevelde, J. Vangeel, N. De Cock, C. Lachat, L. Goossens, K. Beullens, L. Vervoort, C. Braet, L. Maes, S. Eggermont, B. Deforche, J. Van Camp

**Affiliations:** 1 Department of Public Health, Ghent University, 4K3, De Pintelaan 185, 9000 Ghent, Belgium; 2 Leuven School for Mass Communication Research, KU Leuven, Parkstraat 45 –bus 3603, Leuven, Belgium; 3 Department of Food Safety and Food Quality, Ghent University, Coupure links 653, 9000 Ghent, Belgium; 4 Department of Developmental, Personality and Social Psychology, Ghent University, H. Dunantlaan 2, 9000 Ghent, Belgium

**Keywords:** Adolescents, Nutrition, Snacks, Diet, Obesity, Overweight, Learning theories, Conditioning, Dual-system model, App, mHealth, Intervention

## Abstract

**Background:**

As the snacking pattern of European adolescents is of great concern, effective interventions are necessary. Till now health promotion efforts in children and adolescents have had only limited success in changing adolescents’ eating patterns and anthropometrics. Therefore, the present study proposes an innovative approach to influence dietary behaviors in youth based on new insights on effective behavior change strategies and attractive intervention channels to engage adolescents. This article describes the rationale, the development, and evaluation design of the ‘Snack Track School’ app. The aim of the app is to improve the snacking patterns of Flemish 14- to 16-year olds.

**Methods:**

The development of the app was informed by the systematic, stepwise, iterative, and collaborative principles of the Intervention Mapping protocol. A four week mHealth intervention was developed based on the dual-system model with behavioral change strategies targeting both the reflective (i.e., active learning, advance organizers, mere exposure, goal-setting, monitoring, and feedback) and automatic processes (i.e., rewards and positive reinforcement). This intervention will be evaluated via a controlled pre-post design in Flemish schools among 1400 adolescents.

**Discussion:**

When this intervention including strategies focused on both the reflective and automatic pathway proves to be effective, it will offer a new scientifically-based vision, guidelines and practical tools for public health and health promotion (i.e., incorporation of learning theories in intervention programs).

**Trial registration:**

NCT02622165 registrated November 15, 2015 on clinicaltrials.gov.

## Background

The dietary pattern of European adolescents with a high intake of energy-dense low-nutritious foods and a low intake of essential food groups is of great concern [1, 2]. In Flanders, 27 % of adolescents consume sweet snacks on a daily basis, and snacks between meals account for 20–24 % of their total energy intake [3, 4]. A recent study among Flemish 14- to 16-year-olds indicated a higher intake of unhealthy –fat- and/or sugar-rich– snacks (214.4 ± 147.3 g) than healthy snacks (122.0 ± 133.4 g) [5]. Given the association between unhealthy snacking and the development of obesity, dental carries and other chronic diseases during adolescence and later in life [6–8], attention to healthy snacking in adolescents is key.

Based on the bio-psycho-social model [9], the development of an effective health promotion intervention requires a multidisciplinary approach addressing multiple contexts both at the individual and environmental level. Most health promotion efforts to improve dietary behaviors in youth have focused mostly on schoolchildren and their families in enhancing knowledge and changing well-known environmental factors in order to improve dietary behaviors [10, 11]. However, till now these school- and family-based multicomponent interventions have had only limited success in changing adolescents’ eating patterns and anthropometrics [12–14]. Therefore, new approaches to influence dietary behaviors in youth should be considered based on new insights on effective behavior change strategies and attractive intervention channels to engage adolescents.

Michie and colleagues [15] conducted a meta-regression to identify the most effective techniques derived from different behavioral change theories on healthy eating and physical activity interventions and found that interventions combining self-monitoring with at least one other technique derived from the Control Theory (i.e., goal setting, feedback, review goals) of Carver and Scheier [16] were more effective than other interventions. In addition, an innovative in-depth analysis of theoretical explanations for behavior change following interventions (i.e., a comparison between the theoretical basis of an intervention, the delivery in practice = dose delivered, and the intervention receipt = dose received/exposure in participants) by the same authors [17] indicated a clear theoretical difference between intervention delivery and receipt. Participants reported a lower exposure than the dose delivered for all used theoretical methods apart from those based on operant theory. This latter result highlights the important role of operant techniques (i.e., rewarding/positive reinforcement) in behavior change.

Next, also personality theories provide important insight as they focus on differences between individuals. An important gap in intervention research is the effectiveness of interventions that take into account individual differences in personality to promote healthy diets [14]. This is unfortunate as unhealthy eating behaviors, such as the intake of energy dense snacks, are often driven by hedonic motives like eating for the palatability or the reward value of food in the absence of hunger rather than by homeostatic hunger or eating out of biological needs [18, 19]. Reward Sensitivity (RS) is a bio-psychological concept which reflects one’s ability to experience pleasure or rewarding feelings when exposed to positive (appetitive/palatable) stimuli [20] and has been found to play a critical role in eating unhealthy food products, overeating and becoming overweight or obese [5, 21, 22]. During adolescence, RS has an increased influence on behavior as the reward processing peaks while regulative control matures at a slower pace [23]. Taking into account RS in preventive health promotion among adolescents by using reward-based strategies might increase intervention effects in this population. However, little is known about the specific role of RS in food cue reactivity and learning processes to adopt healthy food preferences and healthy nutrition behavior. Therefore, the REWARD-project aimed to develop a new approach to motivate adolescents to opt for healthy snack choices by using reward-based learning and taking into account individual differences in RS.

Although recent insight on the role of learning theories in behavior change has increased considerably, we have to take care not to rely too much on a simplistic perspective. Kremers and colleagues [11] indicated that nutrition behaviors are the result of a joint function between conscious and unconscious processes. Dual-system models or dual-process models explain health behaviors as two interconnected mental systems, each operating according to different principles [24, 25]: a top-down reflective system including elaboration and cognitive efforts to build beliefs and decisions and a bottom-up impulsive/automatic system (i.e., habits) in which certain stimuli or cues are linked to certain behaviors based on earlier learned associations [11, 26]. Based on previous research and theoretical evidence [11, 24–26], it was decided to incorporate behavioral change methods and strategies in the intervention to influence the reflective pathway in addition to the reward-strategies that target the more automatic pathway. This broad framework is attractive as it allows to include other models that focus on unique determinants like personality theory and operant learning models that fit well with the bottom-up perspective as well as the Control theory that focuses more on the reflective pathway.

Finally, besides using new theoretical approaches, it is also warranted to revise the intervention channel to reach adolescents. Traditional channels, via school, family, and/or community, had only limited success in previous studies [12–14]. We therefore assume that intervention approaches imbedded in the actual world of adolescents are needed. Smartphones have a great potential to reach large numbers of adolescents as they have become an integral part of adolescents’ daily life and usage rates are rising significantly in European adolescents [27]. Recent systematic reviews have shown that mHealth interventions are promising in changing health behaviors in youth [28–30]. Additional advantages are the cost-effectiveness of the dissemination, lowered participant burden, flexible program tailoring, data for self-monitoring, and more visually appealing and engaging multimedia modalities [31]. Therefore, it was decided to deliver the intervention as an mHealth intervention.

This paper describes the rationale, design and methods of a four-week controlled trial designed to measure the effectiveness of an mHealth intervention named the ‘Snack Track School’ to improve adolescents’ snacking behavior. The first hypothesis is that the intervention will increase the healthy snack intake and reduce the unhealthy snack consumption (i.e., measured by a healthy snack index) of adolescents compared to the control group. Secondary hypotheses are that the intervention will 1) decrease the age- and gender-adjusted Body Mass Index, 2) positively influence socio-cognitive factors and habits related to healthy snacking, and 3) affect high reward-sensitive adolescents’ snacking behavior more than low reward-sensitive adolescents.

## Method

### Study design and setting

The design consists of a controlled pre‐post design. The intervention will be organized in three secondary schools in one city in Flanders (Belgium), while three schools from a similar (matched) city (comparable socio‐economical characteristics, population density, size) will be selected as a control setting. The adolescents in the intervention schools will receive a four-week mobile intervention while the control schools will continue their usual practices. The full study period will consist of a baseline test, the four-week ‘The Snack Track School’ intervention (or 4 week control), and a post-test immediately after the intervention.

### Study population and recruitment

The study population will consist of 14- to 16- year-old Flemish adolescents (i.e., 3^rd^- 4^th^ grade of Flemish secondary schools). The necessary sample size for this study was calculated based on the healthy snacking index (i.e., the primary outcome of the intervention study). Assuming an intraclass correlation (ICC) of 0.02 at school level and an ICC of 0.03 at class level with a mean and standard deviation of the healthy snacking index of 37.8 ± 20.2, at least 12 classes with 15 adolescents per school (three intervention schools and three control schools) are needed to detect a difference of 20 % between intervention and control at the 5 % significance level with a power of 80 % [32]. The ICC’s, mean and standard deviation regarding the healthy eating index were based on the earlier cross-sectional REWARD study and the test-retest of the REWARD Food Frequency Questionnaire (FFQ) to measure snack intake ([5], De Cock unpublished Data]. To account for possible loss to between pre and post an oversampling of 33 % will be applied. A total sample of 1437 adolescents (control and intervention) in 24 classes per school will be recruited. No exclusion criteria will be applied.

Following the acceptance of the school to participate in the study, parents will receive a letter explaining the purpose of the study and asking for passive consent for participation of their child in the study. In addition, consent of the adolescent will be asked at the beginning of the study.

The study adheres to the Helsinki declaration and the conventions of the council of Europe on human rights and biomedicine. Approval for the intervention study was provided by the Medical Ethics Committee of the University Hospital Ghent and the University of Leuven.

### The ‘Snack Track School’ intervention

#### Intervention development

As earlier mentioned, the ‘Snack Track School’ intervention includes behavioral change strategies to influence both the automatic pathway as well as the reflective pathway. The systematic, stepwise, iterative, and collaborative principles of the Intervention Mapping protocol [33] were used to put this idea in practice. The intervention was preceded by an extensive problem analysis (i.e., needs assessment) using existing and newly generated evidence, including a cross-sectional survey, experimental and focus group research ([5], De Cock Unpublished data).

Following the Intervention Mapping protocol, different stakeholders were involved in the intervention development: adolescents, teachers, principals, stakeholders from food industry, professional organizations active in health promotion, community members, umbrella school organizations. Adolescents were involved in both the conceptual (i.e., a large scale focus group research among 101 adolescents) as well as the pretesting phase of the intervention (i.e., via test labs in smaller groups of four to eight students to regularly gather feedback regarding feasibility, usability, and attractiveness of the app). Monthly teacher contacts (two per participating school) were also included during the process to ensure that the app format was fully compatible with existing pedagogic guidelines, expectations, and school programs. Other stakeholders (i.e., food industry, professional organizations active in health promotion, community members, umbrella school organizations) were frequently consulted through stakeholder meetings to explore if and affirm that the current intervention can be disseminated to the wider society. A strong participation of these actors was used in the different phases of intervention development to ensure a culturally-, age- and community-relevant intervention which might increase the likelihood of program success.

#### Theoretical basis

The dual-system model [11], a model explaining health behaviors as a joint function between conscious and unconscious processes, was chosen as the broad framework of our intervention, as it allows to include other models like personality models, operant learning models and the Control theory. First, the dual model suggests that behavior change has to take into account the bottom-up impulsive/automatic system (i.e., habits) in which certain stimuli or cues are linked to certain behaviors based on earlier learned associations. Moreover, this fits with personality theory that describes a high peak of reward sensitivity in adolescence and its important and understudied role in nutrition behavior. However, this also fits with operant models of behavior change that argue that reward-based strategies (i.e., provision of rewards and positive reinforcement) are needed as the main behavioral change strategies.

**Fig. 1:**
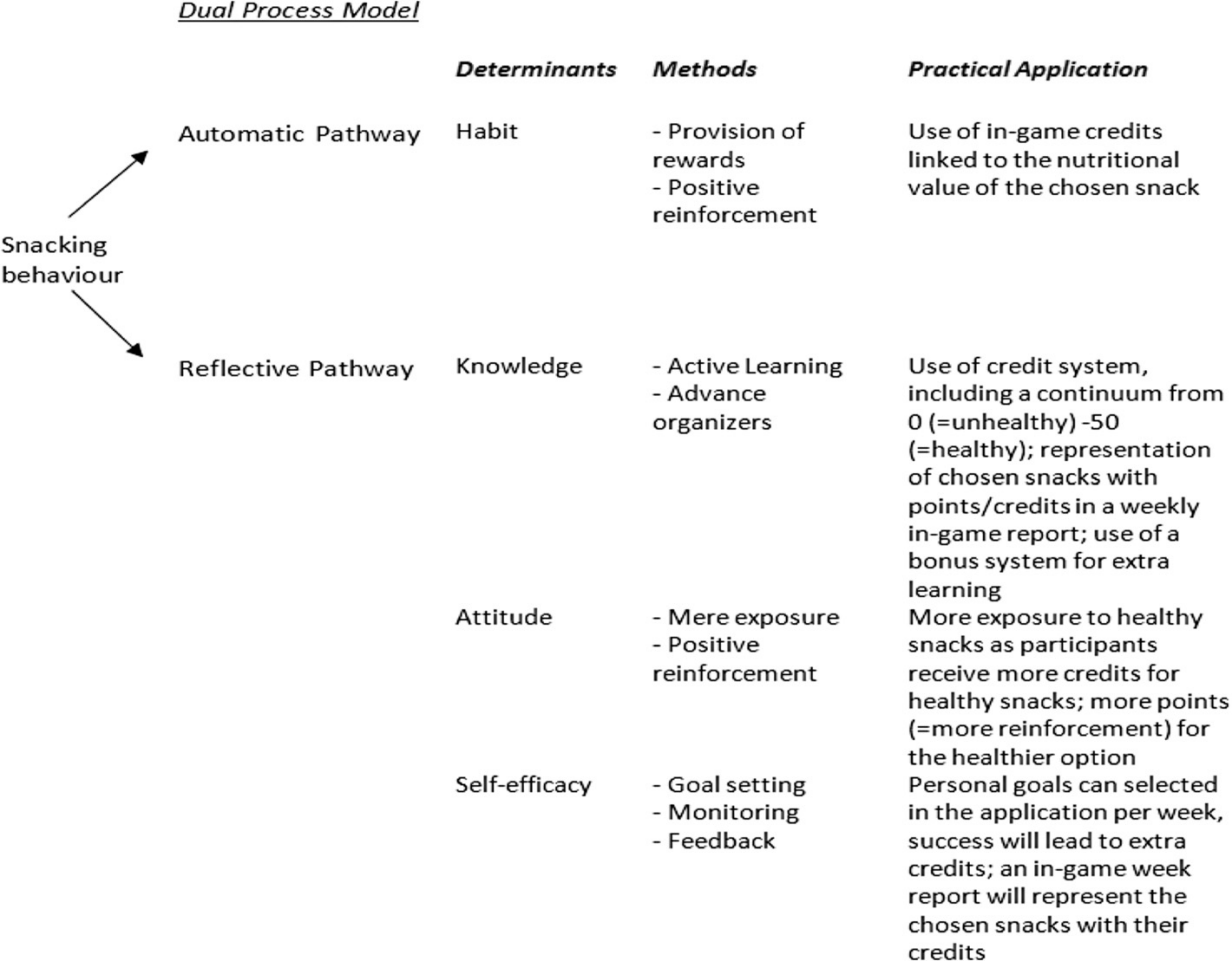
An overview of the theoretical framework of the intervention including the determinants, theoretical methods/behavioral change strategies, and practical applications

However, the model plaids also to include top-down determinants including elaboration and cognitive efforts to build beliefs and decisions. Based on the extensive needs assessment, the following correlates at the individual level were found to be associated with the intake of snacks: knowledge, attitude, and self-efficacy. More knowledge about the healthiness of snacks, more positive beliefs towards healthy snacks, and a higher self-efficacy to eat healthy snacks, were associated with a higher intake of healthy snacks (REWARD unpublished data, [34–37]). These significant cognitive correlates will be targeted in the intervention via behavior change techniques like active learning, advance organizers, mere exposure, goal-setting, monitoring, and feedback to influence the reflective pathway (see also Fig. 1 and below for more details).

#### Intervention components

The ‘Snack Track School’ app is a four week intervention and  constitutes of a virtual high school environment with typical school locations (i.e., classrooms, gym hall, bike area, bathrooms, etcetera) in which all participants have their own locker, their own Snack Track Tool, and their own ‘anonymous’ avatar, which they can customize as preferred based on several options (i.e., many options for head of character, clothes, shoes, gadgets). During the four-week intervention, every week of snack monitoring has its own story line and challenges.

The central idea of the ‘Snack Track School’ intervention is that participants earn credits/points based on the nutritional value of the snacks they consume (i.e., *positive reinforcement/providing rewards to influence automatic behavior*). For every snack the adolescents enter in the app via the ‘Snack Track’ tool, they will be awarded by points. The healthier the snacks they consume, the more points they will receive. These credits will be responsible for the progress in the weekly challenges of the four-week app. No negative points will be provided for unhealthy snacks as punishment can have contra-productive effects [38]. A large snack database was constructed based on the Internubel Trade Name database [39]. Points will be awarded according to the UK Ofcom Nutrient Profile model [40].

In order to stimulate a well-balanced snacking pattern and not merely the tracking of as many snacks as possible, some *gratuities and limitations* are built in the app related to the credit system. Participants are able to track as many snacks as they want, however, only the credits of the first ten snacks are included. Three gratuities are included: 1) a bonus for a snack intake ≤ six snacks per day, 2) a bonus for a snack intake of ≥2/3 healthy snacks per day, and 3) a bonus for non snackers that are involved in the app (logging in ≥3 times in the app per day). The cut-offs of six snacks and more than 2/3 healthy snacks were based on Flemish guidelines of necessary food and nutrient intake [41]. It is advised to consume maximum 10 % energy from snacks (defined here as unhealthy snacks). Fourteen to 16- year-old girls and boys have a general advised total energy intake per day of 2100 and 2600 kcal, respectively [41]. This corresponds with a maximum intake of 210–250 kcal from unhealthy snacks (i.e., one or two unhealthy snacks per day depending on the size and nutritional content). Additionally, it is advised for adolescents to eat three pieces of fruit and four dairy products per day (i.e., not only via snacks but also via meals) [41]. The cut-off of maximum six snacks and more than 2/3 healthy snacks per day was deduced from these guidelines, an intake of three or four healthy snacks together with one or two unhealthy snacks is acceptable for adolescents as they need a lot of energy to grow.

The following theoretical methods were included in the app to affect the reflective pathway including knowledge, attitude, and self-efficacy. Active learning and advance organizers based on the Elaboration Likelihood Model were included to enhance the knowledge of the nutritional quality of a snack item. The Elaboration Likelihood Model [42] suggests that people only process information centrally (i.e., careful consideration which can increase knowledge) when they are motivated to do so, when information is personally relevant, and when the information is presented repeatedly. Through the provision of credits in the app and the progress in the app linked to these credits, participants will unconsciously learn about the healthiness of a snack. The credit system used in the app can be seen as an external motivator to change their snacking behavior. Insights about the (un)healthiness of snack items based on this credit system can stimulate central processing of this information as more knowledge about the specific credits per snack item and the application of this knowledge can enhance their progress in the app. These points are also personally relevant (i.e., linked to the own snacks consumed and related to progress in the app) and are repeatedly presented. In addition, this healthy-unhealthy continuum ranging from 0 to 50 and the placement of the different snack items on this continuum based on the credits, can act as a mental reminder (i.e., a mental axis) regarding the nutritional value of snack items using the method of advance organizers. Furthermore, the earlier mentioned gratuities and limitations also learn adolescents about a well-balanced snacking pattern.

The method of mere exposure is used to activate positive *attitudes* regarding healthy snacks. Zajonc [43] indicated that participants develop a more positive attitude about stimuli following repeated exposure. In our intervention more exposure to healthy snacks is expected as participants receive more credits for the healthy choice, i.e. the expected behavior, which subsequently can lead to increased exposure and thus more liking or positive attitude even if they are not consciously aware of the process [43]. Moreover, the association between positive reinforcement and healthy snacks can also result in more positive attitudes [44].

*Self-efficacy* will be targeted via goal setting, monitoring, and feedback. Goal setting has a high likelihood to improve health behaviors because persons who set goals exert themselves to a greater extent, persevere in their aims, concentrate more, and develop strategies to be able to perform the necessary behavior [16, 45]. Adolescents will choose a goal per week regarding improving their snacking habits. The chosen goal will be evaluated per day and extra bonus credits/rewards will be provided in case of success. At the end of every week, *feedback* will be provided via a week-report that portrays all consumed snacks per weekday with their matching credits. This week-report will also provide feedback about the fail or success of reaching their goal. In case of success (i.e., linked to the goals), it will increase self-efficacy/capability of eating more healthy snacks. In case of failure, the feedback and the pending reward can stimulate elaboration and another attempt, and a possible increase in self-efficacy after success in the future [16]. *Goal setting* will be applied from week 2 till week 4. At the beginning of these three weeks, participants will be provided four goals. They need to choose one specific week goal, which they need to reach every day during that week. In case of success, a bonus of 150 points will be won at the end of the day. Earlier research has indicated that *self-monitoring* is the most successful behavioral change technique in energy balance-related intervention research [15]. Participants need to enter every consumed snack in the app and can monitor their snack intake in a weekly report. Based on the self-monitoring, they will have more insight into and awareness of their snacking behavior. This week-report can create elaboration about the snacking behavior.

To increase participants’ motivation to play our app, the most significant game motivations and dynamics based on the cross-sectional REWARD study, focus group research and previous studies (REWARD unpublished data, [46–48]) were included. *Challenge* (i.e., to push yourself to a higher level of skill or personal accomplishment), *competition* (i.e., proving who has the best skills and can react and think the fastest), and *social interaction* (i.e., playing together with others both online as offline) were found to be important motivations for adolescents to play games. Furthermore, in the cross-sectional REWARD study, adolescents indicated the following characteristics as most crucial: (1) competitive aspects/playing against other people/leader board rankings, (2) cooperation/working together to reach goals, (3) different story outcomes based on your player actions, (4) ‘levelling up’ a game character, and (5) earning points or other rewards (unpublished data). Earlier studies also highlighted the importance of cooperation, and rewards as game dynamics [46–48]. Baranowski and colleagues [49] and Peng [50] indicated the importance of story lines to increase immersion and intrinsic motivation to play games. Moreover, the inclusion of three psychological needs based on the Self-Determination Theory [46, 49, 50], namely autonomy, competence, and relatedness, have been linked to increased intrinsic motivation to play games. From this viewpoint, it is necessary that participants can make their own choices in a game that lead to different endings (i.e., autonomy), can customize their own avatar according to their preferences (i.e., autonomy and relatedness), have indicators for their achievements as this better satisfies players’ needs of competence.

### Implementation and evaluation of the intervention

The four-week ‘Snack Track School’ app will be evaluated via a matched controlled pre‐post- design including a baseline test and post-test immediately after the end of the intervention. The app will be launched via the schools but will not be imbedded in the school curriculum, the app is a stand-alone intervention that can be used by the adolescents without involvement of the teachers. The intervention will be conducted in the spring of 2016.

Weekly process evaluation moments will provide insight into participation of adolescents in the app and will also be used as a moment to encourage participation. During the study period, teachers and students will be able to contact the researchers by phone or email. If necessary, visits of the researchers will be possible in addition to the weekly evaluation moments. In addition, a help function is built in the app so participants can always contact the researchers and game developers in case of technical or other problems. Smartphones (Nokia Lumia 435) will be provided to the adolescents without (functional) smartphone so all adolescents in the intervention schools can participate.

### Data collection and outcome measurements

#### Primary outcome measure

The primary outcome consists of the *Healthy Snacking Index*. A Food Frequency Questionnaire (FFQ) was developed to measure average snack consumption in adolescents via 28 snack items. This new FFQ is based on an earlier FFQ instrument for children developed by Huybrechts and colleagues [51], but includes portion sizes that match adolescents’ intake patterns. Adolescents will indicate their answer in a list of six frequency categories, namely never or seldom; 1–3 days/month; 1 day/week; 2–4 days/week; 5–6 days/week; every day [51]. The FFQ contains four to six daily portion size categories per snack item and a list of common standard measures as examples. Snacks are in this study defined as all food items consumed outside (>30 min) of breakfast, lunch and dinner, in accordance to Rodriguez and Moreno’s definition of snacking [52]. The 28 snack items assessed in the FFQ are: dried fruit, fruit, raw vegetables, nuts and seeds, chocolate and pralines, candy bars, candy, dry cookies, other cookies such as chocolate cookies, breakfast rolls, pastries, breakfast cereals, unsweetened yoghurt, sweetened yoghurt, pudding, mousses, ice-cream, popsicles, sandwiches with sweet or savoury spread, cheese or meat cubes, chips and similar products, other savoury snacks such as bread sticks, sausage/cheese rolls and pizza, other fried snacks such as spring rolls and cheese croquettes, fries, kebab, hamburgers and pasta cups.

The classification of snacks and drinks into healthy and unhealthy was based on the nutrient profiling model as developed by the UK NP Ofcom model [40]. This model calculates for each food product a score that represents its healthiness In order to apply this model, an average nutrient composition per FFQ category was needed to apply this nutrient profile model. The average nutrient composition per FFQ category was calculated by averaging the nutritional composition (obtained from the Belgian food composition table [39] expressed per 100 g) of the most frequently consumed food items by adolescents within that category, as reported in the HELENA study [53]. The average energy, sugar, and fat and sodium intakes per FFQ category were then calculated by multiplying the amounts (g) of the food consumed and the average nutritional values expressed per g (the average values per 100 g divided by 100) [39]. According to the average amount of sodium, sugar, fat and kJoules, fibre, proteins and the portion of fruit and vegetables or nuts present in the product the NP model gives the food product a score, if a food product scores more than 4 points it was considered unhealthy. The FFQ items crisps, other salty snacks, sausage/cheese rolls and pizza, other fried snacks, fries, hamburgers, cheese or meat cubes, icecream, popsicles, breakfast cereals, pudding, sandwiches with sweet or savory spread, mousses, chocolate, candy bars, candy, dry cookies, other cookies, breakfast rolls and pastries were considered to be unhealthy.

The daily intake of each FFQ category will be obtained by multiplying the frequency of consumption with the quantity of consumption per week (g) divided by 7. These daily intakes, will then be summed to obtain the daily intake of healthy snacks (g), and unhealthy snacks (g). Finally a health index for snacks will be calculated: (gram healthy snacks/‘gram healthy snacks + gram unhealthy snacks))*100. The reliability and validity of this FFQ to assess the healthy snack ratio for interventions purposes were tested and reported elsewhere [De Cock unpublished]. The healthy snack index’ reliability was good and the validity was acceptable.

#### Secondary outcome measure

##### Anthropometry

Two trained research assistants will measure body height and weight according to a standardized protocol. Adolescents will be measured without shoes and will be allowed to wear light clothing, such as a t-shirt and shorts/short pants. Body height will be measured with a Seca Leicester Portable stadiometer with an accuracy of 0.1 cm. Weight will be measured with a calibrated electronic scale SECA 861 with an accuracy of 0.1 kg. Two readings of each measurement (weight and height) will be obtained to assure accuracy. If the two readings differ more than 1 %, a third measurement will be taken. These three measurements will be recorded and the outlier will be excluded during the data cleaning process.

##### Cognitive variables

As reward-based strategies are combined with goal setting, self-monitoring, active learning, and advance organizers, effects on the following cognitive variables can be expected [43–45, 54]. All constructs, apart from habit and knowledge, were based on the reliable and valid Healthy Diet determinants of the HELENA study [55]. Additionally, indirect effects of this intervention can be expected on peers’ modelling and social support and pressure given the chosen game motivations and dynamics in the app (i.e., competition and corporation).

- *Awareness* about the healthiness of the adolescents’ snacking and *intention to change the snacking behavior within the next six months* will be assessed using one question with a five-point answer format.
- *Attitude* will be measured with five items in which adolescents’ opinion will be asked on statements linking healthy snacks to taste and health.
- *Self-efficacy* will be assessed via three items asking adolescents how hard it is to eat healthy snacks in general and in two more specific situations (at home, and at school).
- *Habit* will be assessed by a four-item automaticity subscale (the ‘Self-Report Behavioral Automaticity Index’ [56]) based on the twelve-item Self-Report Habit Index (SRHI) [57]. This subscale was found to be reliable and sensitive to detect the habit-behavior association and moderation of the intention-behavior relationship in energy balance-related behavior domains [56].
- *Knowledge* about the healthiness of specific snacks will be measured by means of a scoring test. Adolescents will be asked to rate the healthiness of each FFQ item (28 in total) by giving it a score ranging from 0 to 100. Zero represents “very unhealthy” and 100 “very healthy”. Afterwards the results will be compared with the actual score of the 28 snack items calculated by means of the UK NP Ofcom model [40].
- *Perceived peers’ snacking behavior, peers’ social support, social pressure, and subjective norm* regarding healthy snacks will be measured by valid and reliable items based on the HELENA and ENERGY study [55, 58].

*Other measurements for explorative research including mediators and moderators*

### Socio-demographics

Adolescent characteristics, including gender, date of birth, ethnicity, and family status, socio-economic status of the family, via the occupational social class of father and mother and the Family Affluence Scale (FAS) [59] will be assessed.

Table 1 provides an overview of all other measurements included in the questionnaires.

**Table 1:** Overview of other measurements for explorative research

Environmental variables/determinants related to dietary behaviors	
Snack availability at home	The home availability of the 28 snack items used in the FFQ. The constructs of these questions are based on valid questions from the European HELENA study [1].
Peer influence	Perceived peers’ snacking behavior, peers’ social support, social pressure, and subjective norm regarding healthy snacks. These items are based on valid and reliable items from the HELENA and ENERGY study [55, 59].
Parental influence	Parents’ modeling, rules at home, and monitoring in relation to snacks. The items are based on valid and reliable items from the HELENA and ENERGY study [55, 59].
Personality traits/biological factors	
Reward sensitivity and punishment sensitivity	The Dutch child version of the Carver and White’s Behavioral Inhibition System (BIS)/Behavioral Approach System (BAS) - scale as developed by Franken and colleagues [62]. The convergence and discriminant validity as well as the internal consistency of the BIS/BAS scale have been demonstrated [63–65].
Restraint	The five-items subscale *restraint* from the Child Eating Disorder Examination Questionnaire (ChEDE-Q [66]). Research demonstrated the reliability and validity of the ChEDE-Q for examining eating pathology in youngsters from the general population [67] and in clinical samples of treatment seeking obese youngsters [68].
Pubertal status	The Pubertal Development Scale [69] which is a five-item self-report questionnaire to ascertain pubertal status in adolescents.
Other measurements on nutrition behaviors	
Total energy intake per day	An FFQ to estimate total dietary intake was developed and has been used in and adjusted for several target population from pre-schoolers to older women [51, 70, 71].
Adolescents’ meal patterns:	The frequency of eating breakfast, lunch and dinner, the frequency of snacking, and the source from which they usually obtain the snacks. All questions originate from the validated questionnaires of the European HELENA study [72].
Game behavior-related variables	
Duration and frequency of game play	Duration will be measured via a timeline separately for each day of the week. F*requency* will be assessed by using a 7-point Likert scale from ‘almost never’ to ‘almost every day’. Respondents are asked to indicate how often they play games on any device. Both methods have been used in previous studies to measure game use [73].
Preferences for game genres	This will be measured separately for *computer/console* games and *smartphone/tablet* games. For both categories a list of 13 game genres has been developed based on previous studies and current popular game genres [74, 75]. Given that new games and even new game genres arise every year, the list was updated to the current situation.
Game motivations	The Uses and Gratifications Questionnaire for game developed by Sherry et al. [76].
Game engagement	The Game Engagement Questionnaire of Brockmeyer and colleagues [77]. This validated instrument consists of 19 items from which a total Game Engagement Score can be derived in order to examine one’s involvement in games.
Game addiction	A 7-item Game Addiction Scale [78] of which each item corresponds with one of the seven criteria for pathological gambling according to the DSM: salience, tolerance, mood modification, withdrawal, relapse, conflict and problems.
Smartphone and tablet use	Respondents will be asked to indicate whether they use a smartphone or tablet (yes or no). Given that our serious game can be played on either of these devices it is important to take into account previous experience with the devices.
Structural characteristics	Adolescents will be asked to indicate how important several structural characteristics of video games are for their game experience. King, Delfabbro & Griffiths [79] recently developed a list of game dynamics based on psychological structures. The researchers have analyzed games and made an overview of game dynamics that are engaging to game players, which they tested on a broad sample of various age groups.

#### School management questionnaire

At the pretest, the principal of every school will be asked to complete a questionnaire assessing the food policy at school. This school management questionnaire is based on the school management questionnaire of the ENERGY project [60]. Since the REWARD project only focuses on healthy food choices only the parts of ENERGY questionnaire that were related to food facilities are used. The questionnaire comprises of the following sections: general characteristics of the school, the physical environment (opportunities to eat and drink at school), the political environment concerning regulations and practices pertaining to food and drinks and a last section specifically on vending machines (typical sale of snacks and soft drinks).

#### Process evaluation

The purpose of the REWARD process evaluation is to describe 1) the reach of the intervention (incl. recruitment of sample); 2) degree of dose delivered by researchers (limited in our intervention as we provide one app to all participants) and dose received (exposure and satisfaction) by adolescents; and 3) contextual factors that may have influenced dose delivered and/or dose received; in order to get insight into intervention effects. Table 2 presents an overview of the measurements of different process evaluation concepts.

**Table 2:** Overview of  the measurements of the different process evaluation concepts

Concept	What/how to measure
1a) Reach (participation rate) Description of who participated and who did not	Schools - describe by type and composition (gender, SES, ethnicity) Adolescents - gender/age/school type/SES Drop-outs - Description of those participating and those not
2a) Dose delivered (completeness) Implementation of all components	Before the start of the intervention, a presentation will be given to the involved teachers and principals to inform them about the intervention and the weekly process evaluation moments. A weekly process evaluation moment will be conducted in the classroom by the teachers or researchers depending on schools’ and teachers’ preference. During this weekly moment, a classical discussion will be held to evaluate the previous game week, to ask about possible problems, to introduce next week’s week story/challenge and to highlight the importance of continuing with the intervention/game. Weekly logs will be held to capture this process.
2b) Dose received (exposure) Participants engagement, interaction, initial and/or continued use	Dose received (exposure) will be measured via two ways: 1) the core game module of the game experience questionnaire that will be included in the post-test questionnaire, and 2) the in-game logs.
2c) Dose received (satisfaction) Satisfaction with intervention	Dose received (satisfaction) will also be measured via the game experience questionnaire.
3) Fidelity Implemented as planned.	Researcher weekly logs - summary of comments/open ended questions on weekly logs
3) Context Physical/social/political contextual factors that might influence implementation/reach	School management questionnaire to describe differences in school systems/organizations and priority of nutrition Teacher post intervention questionnaire, Principle post-test, and question in adolescent posttest questionnaire on school related factors likely to influence implementation/contamination

During the intervention, weekly process evaluation moments (15 min) will be included in the classroom, these will be implemented by the researchers and will be audiotaped. A process evaluation questionnaire will be added to the adolescent questionnaire at the post-test to assess participants’ engagement and use of the app, and the interaction between peers resulting from playing the app. In addition, adolescents’ dose received and satisfaction will also be assessed through logs of the app. Contextual factors will be measured through a post-test questionnaire for adolescents, the principals of the schools (both control and intervention) and involved teachers.

#### Data management and analysis

After every data collection wave (baseline, post), adolescent questionnaires will be scanned using the scanning software package TELEForm (version 6.1, Cardiff Software Inc., San Marcos, California, USA). Afterwards data will be translated into an SPSS file.

Descriptive analyses will be used at the individual level to compare the intervention and control group at baseline. Linear mixed models taking into account the clusters (child-class-school) will be used to evaluate differences in the primary and secondary outcome variables. In addition, subgroup analyses, and mediating and moderating analyses will be conducted to provide more insight into intervention effects. The process data (including objectively measured data and self-reported data) will be translated in a variable indicating the degree of intervention implementation and will be linked to the effect evaluation.

## Discussion

The newly developed gamified ‘Snack Track School’ app tries to tackle some of the problems identified in earlier prevention programs as these previous interventions were not successful in changing nutrition behaviors and anthropometrics [12–14]. Previous nutrition interventions were often based on providing general information: all participants received comparable information on nutrition and eating behavior, irrespective of their individual differences. Since interventions taking into account individual differences are expected to have a higher likelihood of being effective in establishing sustainable behavior change [14, 61], the ’Snack Track School’ app aims to motivate adolescents to opt for healthy snack choices by using reward-based learning and taking into account individual differences in RS.

During the development process of the ’Snack Track School’ all the possible intervention success factors based on own and earlier research were gathered, i.e., an intervention focusing on both the automatic and reflective pathway using significant behavior change methods and techniques and an attractive intervention channel for adolescents. By describing RS as a determinant of nutrition behavior and food choices in adolescents and combining this with reward-based learning paradigms in the intervention, we believe an innovative framework is introduced in public health interventions. If this paradigm shift would turn out to be successful, it will offer a new scientifically-based vision, guidelines and practical tools that stakeholders can use to move to better dietary habits in youth.

## Abbreviations

FFQ, food frequency questionnaire; RS, Reward sensitivity
